# Evaluating inner retinal dimples after inner limiting membrane removal using multimodal imaging of optical coherence tomography

**DOI:** 10.1186/s12886-018-0828-9

**Published:** 2018-06-27

**Authors:** Jingjing Liu, Yiye Chen, Shiyuan Wang, Xiang Zhang, Peiquan Zhao

**Affiliations:** Department of Ophthalmology, Xin Hua Hospital, Shanghai Jiao Tong University School of Medicine, Shanghai, 200092 China

**Keywords:** Inner limiting membrane peeling, Inner retinal defects, Macular hole, Optical coherence tomography, Vitreoretinal interface

## Abstract

**Background:**

To evaluate inner retinal dimples after peeling of the inner limiting membrane (ILM) for macular holes (lamellar macular hole [LMH] and full-thickness macular hole [FTMH]) via multiple imaging modes of spectral-domain optical coherence tomography (OCT) and to assess their relationship with preoperative vitreoretinal interface conditions.

**Methods:**

The data of 38 eyes in 35 patients who underwent surgery for LMH, and FTMH were retrospectively studied. The presence of postoperative inner retinal dimples was judged by a combination of en face OCT layer images and cross-sectional images. The demographic and clinical characteristics of eyes with and without inner retinal defects were compared to identify factors involved in the formation of the defects.

**Results:**

Inner retinal defects were found in 26 eyes (68%) after surgery. They appeared on the en face OCT ILM layer images as multiple dark spots limited to the ILM peeling area, and corresponded to dimples or pitting of inner retinal layers on cross-sectional OCT images. In 5 cases (19%), apparent progression of inner retinal defects was observed on the en face OCT images as increasing numbers and sizes of the dark spots, which seemed to follow an eccentric growth pattern starting from the central macula. In addition, highly myopic eyes were found to be associated with the formation of more severe inner retinal defects.

**Conclusions:**

Multiple imaging modes of en face spectral-domain OCT provide comprehensive information about the appearance of inner retinal dimples. High myopic eyes seem to develop more severe inner retinal defects after ILM peeling.

## Background

Tadayoni et al. [1] first found inner retinal defects after epiretinal membrane (ERM) removal in 2001. They reported findings of a fundus with an appearance characterized by dark arcuate striae along the course of the optic nerve fibers, and called it dissociated optic nerve fiber layer (DONFL). To date, inner retinal defects have been reported to occur in patients after inner limiting membrane (ILM) peeling, which is performed for various indications including ERM, retinal vein occlusion, diabetic macular edema, and macular hole [2–10]. The incidence of these defects seems to be higher in cases with a macular hole (MH) [7]. To the best of our knowledge, inner retinal defects were not frequently evaluated via en face OCT imaging, which can provide not only B-scans but also en face C-scans of the different retinal layers [5, 7, 10]. On ILM layer images, we can identify inner retinal abnormalities more readily, and view their correspondence with the cross-sectional OCT images. This peculiar appearance has not been associated with compromised visual function to date [6, 8].

Since this discovery, inner retinal defects after ILM peeling have been associated with a DONFL appearance [1], concentric macular dark spots (CMDS) [5], and inner retinal dimpling [6]. Appropriate nomenclature for these defects is yet to be determined as their etiology remains unclear. Injury from surgical manipulation [1, 6], ILM removal [3, 4, 8], intraoperative ILM staining with dyes, and gas tamponade [9] are proposed risk factors for apparent inner retinal defects, with ILM peeling considered to be the main culprit. To date, other preoperative clinical factors related to the surgical condition have not been evaluated with respect to their relationship to the formation of inner retinal defects. Since inner retinal defects represent postoperative retinal surface changes after surgery, we assessed the preoperative vitreoretinal interface conditions (lamellar hole-associated epiretinal proliferation [LHEP]), ERM, and high myopia-associated posterior staphyloma) between eyes that developed inner retinal defects and those that did not, to identify the predisposing factors for inner retinal defect formation. LHEP is demonstrated on OCT as a thick homogenous layer of material with medium reflectivity on the epiretinal surface [11–13]. It occurs in both lamellar macular hole (LMH) and full thickness macular hole (FTMH). Unlike the conventional ERM, LHEP does not appear to have contractive properties [11]. The LHEP appearance is primarily driven by a proliferation of Müller cells onto the inner retina [13]. MHs complicated by ERM and LHEP might cause changes such as an imbalance of damaged and regenerative glial cells both before and after ILM peeling, which influences the formation of inner retinal defects. Khun [14] reported that rigidity of the ILM was responsible for the development of an MH in highly myopic eyes, and even observed the separation of the ILM from the retina within the bulging area of one eye with posterior staphyloma. Hence, the connection between ILM and the underlying retina in eyes with posterior staphyloma was assumed to be different from that in eyes without posterior staphyloma. Accordingly, the impact of ILM peeling on the inner retina was different from a chronic separation between the ILM and retina in eyes with posterior staphyloma, especially with ILM detachment. We also reviewed other general features such as age, sex, laterality, type and size of MH, and intraoperative findings (ILM peeling area, hemorrhagic spots), that may be causative factors for inner retinal defect formation.

## Methods

We retrospectively reviewed the data of 138 consecutive cases of patients with MHs who underwent pars plana vitrectomy (PPV) with ILM peeling between January 2013 and October 2017. Patients who had successfully undergone PPV with ILM peeling for MHs were regularly evaluated for the postoperative macular conditions using high quality en face and B-scan imaging for at least 6 months. Patients who lacked the en face OCT images for analysis or had other notable retinal conditions that limited visual acuity, such as myopic choroidal neovascularization, diabetic retinopathy and age-related macular degeneration, were excluded from the study. Finally, 38 eyes in 35 patients were included in this study. Informed consent was obtained from all patients. The study protocol was performed in accordance with the Declaration of Helsinki, and was approved by Ethics Committee of Xin Hua Hospital affiliated to Shanghai Jiao Tong University School of Medicine.

All surgeries were carried out by the same experienced surgeon (P.Z). The surgical procedure consisted of a standard, three-port PPV, with induced posterior vitreous detachment if the posterior hyaloid was attached. The peripheral vitreous was then removed as much as possible, and the peripheral retina was checked circumferentially. Laser photocoagulation was applied to if any retinal tears or lattice were detected. ILM was removed followed by a pinch-and-peel technique in a circular manner with the end-gripping forceps under the assistance of brilliant blue G. The ILM peeling area was decided by the surgeon according to the hole’s diameter. The ILM peeling area and hemorrhagic spots during ILM peeling of each patient were documented. At the end of surgery, air-fluid exchange was performed with gas tamponade by 15% perfluoropropane (C3F8). In cases where the patient presented with LHEP, the yellow tissue was not removed forcefully from the edge of the hole for fear of triggering further damage. Concomitant cataract operations were performed when a combined procedure was planned.

A preoperative and postoperative ophthalmic examination, including measurement of best corrected visual acuity (BCVA) and intraocular pressure, slit-lamp biomicroscopy, dilated fundus examination, ultrasonography and optical coherence tomography (RTVue XR100–2, Optovue Inc., Fremont, CA, USA), was performed on all the study eyes. OCT scans performed before the surgery and at 1, 3, and 6 months after surgery were analysed. The OCT scan modes included radial lines (12*9 mm), horizontal lines (12*9 mm), 3-dimensional retina (7*7 mm), and 3-dimensional Widefield Motion Correction Technology (12*9 mm). Simultaneously, reference images were obtained to observe the thickness distribution of the retina. The diameter of the FTMH was measured as the largest diameter of the 12-radial-line scan pattern centered on the MH. Patients with poor-quality en face OCT images due to poor segmentation performance (some patients with high myopic eyes) were excluded. Microperimetry was performed in 12 patients both preoperatively and postoperatively using the CenterVue MAIA (CenterVue, Padova, Italy). The testing mode was 4–2 strategy: 37 test loci arranged in a radial pattern covering the central 6° region of the retina. The following features were recorded: age, sex, laterality of eyes, cause (s) and type of MHs, size of FTMH, presence or absence of LHEP and ERM, and posterior staphyloma associated with high myopia, anatomic outcome, and functional outcome. To distinguish inner retinal dimples from the inner retinal corrugations caused by other conditions such as ERM, en face in combination with cross-sectional OCT images were used to diagnose the presence of inner retinal dimples. All the postoperative images were evaluated by 3 individual examiners and divided into 2 groups: eyes with inner retinal dimples, and eyes without retinal dimples according to the presence of dimples (≥1) of the inner retinal layer and corresponding dark spots found on en face ILM images. The demographic and clinical features of the two groups were evaluated and compared using statistical analysis. The distribution patterns of the inner retinal defects shown on en face OCT images were analyzed.

The SPSS software, version 22.0 (SPSS, Inc., Chicago, IL), was used for all statistical analysis. The binary variables were compared using the chi-square test or Fisher’s exact test, and continuous variables were compared using student’s t-test and analysis of variance. Logistic regression analysis was used to analyze the risk factors in inner retinal dimple formation. Preoperative and postoperative BCVA were converted into the logarithm of the minimum angle of resolution (logMAR) for statistical analysis. Counting fingers and hand motions were replaced by a decimal visual acuity of 0.014 and 0.005, respectively, before conversion. A *p* value of < 0.05 was considered to indicate statistical significance.

## Results

Thirty-eight eyes of 35 patients with a mean age of 61 years (range: 10–80 years) were included in this study. There were some hemorrhagic spots caused by superficial retinal capillary breakage after ILM peeling. However, no hemorrhagic spots due to direct surgical manipulation were found. Twenty-eight (80%) of the patients were women. The mean follow-up time was 8.7 months. The MH involved the right eye in 17 patients (49%), the left eye in 15 (43%), and both eyes, in 3 (9%). The preoperative diagnosis included 8 LMH (21%), and 30 FTMH (79%). The etiology of the MHs was idiopathic in 36 patients (95%), and traumatic in 2 (5%). Anatomical closure was achieved in all eyes (100%) at the last follow-up. The mean preoperative visual acuity was 1.01 logMAR, which was significantly improved to 0.63 logMAR after surgery (*p* < 0.001). Preoperative, postoperative and changes in BCVA between the two groups were not significantly different (*p* = 0.693, 0.968, and 0.679, respectively). The mean preoperative and postoperative average sensitivity thresholds in microperimetry were 21.70 dB and 25.10 dB, respectively (*p* = 0.095). Microperimetry was performed in 9 eyes with inner retinal dimples and in 3 eyes without inner retinal dimples. Preoperative, postoperative and changes in average sensitivity thresholds between the groups were not significantly different (*p* = 0.430, 0.633, and 0.430, respectively).

**Fig. 1:**
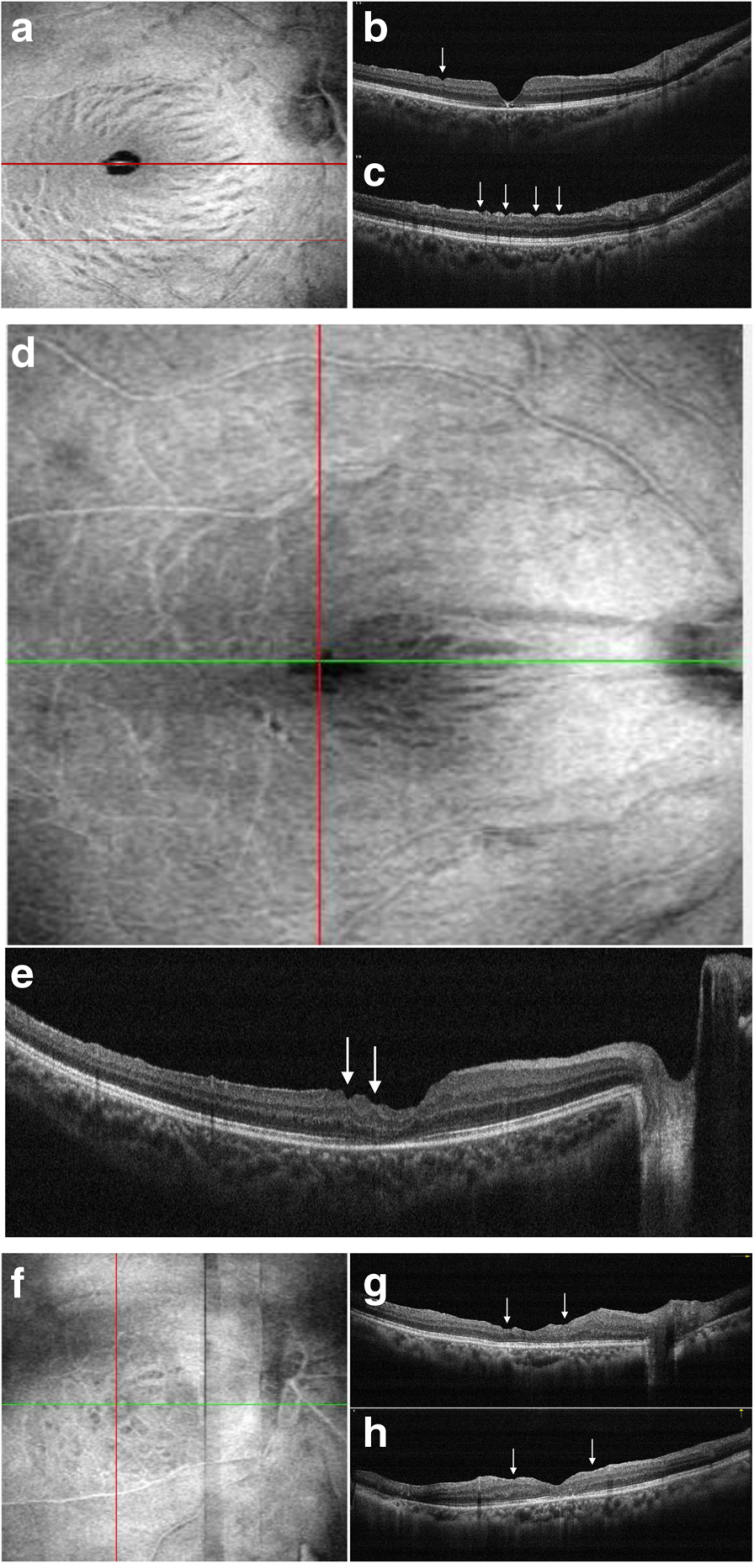
Three distribution types of inner retinal defects on en face OCT ILM layer images **a** Dark striae run along the nerve fiber path and spared temporal raphe area; **b** and **c** corresponding cross-sectional OCT images show pitting of nerve fiber layer (arrows); **d** dark striae were found predominantly occurred in papillomacular bundle area; **e** corresponding B-scan OCT image shows pitting of nerve fiber layer, thinning of underlying ganglion cell layer is also noted (arrows); **f** multiple dark spots with different sizes scattered on the ILM peeling area and seem to not run along the nerve fiber path; **g** and **h** corresponding cross-sectional OCT images show significant thinning and disorganized of inner retinal layers (arrows)

**Fig. 2:**
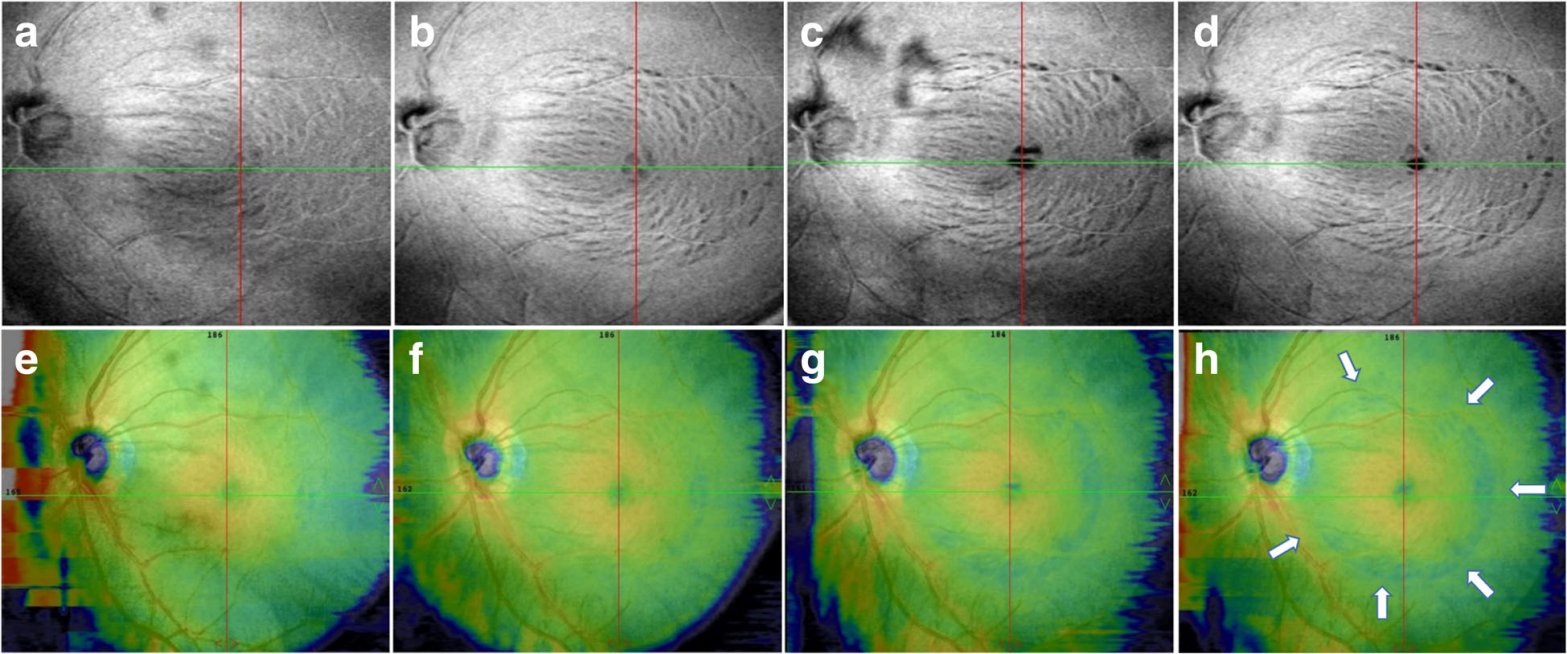
En face OCT images show progression of inner retinal defects after ILM peeling **a** Inner retinal defects were found on en face OCT ILM layer image 1 month after surgery; **b** 3 months after surgery, inner retinal defects showed progression with increasing numbers of dark striae compared with the previous OCT documentation; **c** 6 months after surgery, progression of inner retinal defects was demonstrated by darker striae and some of them were even confluent with each other; **d** 15 months after surgery, inner retinal defects demonstrated similar appearance with last OCT image except for the disappearance of a previous dark spot lying superior of fovea; **e**, **f**, **g**, **h** corresponding reference images show ILM peeling area distinctly (marked by arrows)

Twenty-six eyes (68%) developed inner retinal defects, as seen on the postoperative OCT images at the last follow-up. On en face OCT ILM layer images, inner retinal defects were identified as multiple dark spots, limited to the ILM peeling area and not corresponding with the hemorrhagic areas during surgery. Preoperative en face OCT images were available for 24 eyes, and no similar appearance was found. There were 3 distribution types (Table 1): in 17 eyes (65%), the dark striae ran along the nerve fiber and spared the temporal raphe area (Fig. 1a); in 3 eyes (12%), the dark striae predominantly occurred in the papillomacular bundle area (Fig. 1d); in 6 eyes (23%), multiple dark spots were scattered about the ILM peeling area, and seemed not to run along the nerve fiber path (Fig. 1f). On cross-sectional OCT images, dimples of the inner retinal surface were discovered that corresponded to the striae, and areas with dots, representing the focal thinning of the underlying retinal nerve fiber layer (RNFL) (Fig. 1b, c, e, g, and h). The focal thinning of inner retinal layers seemed more severe in the multiple dark spots than in the other two spots (Fig. 1g, h). No similar appearances were found among the preoperative en face and B-scan OCT images. In 5 cases (19%), we observed an apparent progression, which appeared on the en face, ILM layer images, as increasing numbers and sizes of the dark spots (Fig. 2). The inner retinal defects started from the central macular and spread to the surrounding area. In 8 cases (21%), the ILM peeling area could be distinguished clearly on the en face layer images or the reference images (Fig. 2).

**Table 1:** Number of Eyes with 3 Patterns of Inner Retinal Dimples After Inner Limiting Membrane Peeling

Categories of pattern	Number of eyes	%
1 Scattered	6	23.1%
2 Papillomacular bundle area	3	11.5%
3 Temporal raphe area-spared	17	65.4%

The associations between the development of inner retinal dimples and the demographic and clinical characteristics are summarized in Table 2. Unfortunately, we did not find any preoperative markers that predicted the development of DONFL (Table 3). The associations between the different categories of inner retinal dimples and preoperative vitreomacular interface conditions and ILM peeling area are summarized in Table 4. We found that eyes with “scattered” inner retinal defects had longer axial length and a higher incidence of posterior staphyloma.

**Table 2:** Comparison of Demographic and Clinical Characteristics in Eyes With and Without Inner Retinal Dimples

	Eyes with Inner Retinal Dimples (*n* = 26)	Eyes without Inner Retinal Dimples (*n* = 12)	*p*
Age (years)			0.931
Mean ± SD	60.5 ± 13.7	60.9 ± 13.5	
Gender			0.453
Women	18	10	
Men	8	2	
Laterality of MH			0.734
Right	13	7	
Left	13	5	
Type of MH			0.232
LMH	4	4	
FTMH	22	8	
Size of MH (μm)			0.844
Mean size±SD	575.55 ± 276.03	598.25 ± 278.82	
Number of MAIA			
Posterior staphyloma			1.000
Yes	3	1	
No	23	11	
LHEP			0.714
Yes	7	4	
No	19	8	
ERM			0.296
Yes	8	6	
No	18	6	
ILMP area (PD)			
Mean ± SD			
Preoperative BCVA			0.693
(logMAR)	1.03 ± 0.48	0.96 ± 0.52	
Mean ± SD			
Postoperative BCVA			0.958
(logMAR)	0.63 ± 0.27	0.63 ± 0.38	
Mean ± SD			
Preoperative Aver.ST (dB)			0.430
Mean ± SD	23.71 ± 3.52	15.67 ± 14.17	
Postoperative Aver.ST (dB)			0.633
Mean ± SD	25.42 ± 3.98	24.13 ± 3.70	

*MH* Macular hole, *LHEP* Lamellar hole associated epiretinal proliferation, *ERM* Epiretinal membrane, *BCVA* Best corrected visual acuity, *Aver.ST* Average sensitivity threshold

**Table 3:** Logistic Regression of Factors Associated with Formation of Inner Retinal Dimples after Inner Limiting Membrane Peeling

Variable		B	S.E.	Wald	Significance	Exp (B)
	Age	−0.025	0.032	0.611	0.434	0.975
	Sex	−1.388	1.197	1.346	0.246	0.250
	Laterality	−0.615	0.850	0.523	0.470	0.541
	Type	0.069	1.324	0.003	0.958	1.072
	LHEP	0.154	1.063	0.021	0.885	1.167
	Refraction	0.003	1.394	0.000	0.998	1.003
	ERM	−1.576	1.212	1.691	0.193	0.207
	Constant	5.535	7.380	0.563	0.453	253.481

**Table 4:** Association between Preoperative Vitreomacular Interface Conditions and Inner Retinal Dimples Formation

Categories of pattern	1	2	3	*P*
AL	27.09 ± 4.47	25.09 ± 1.64	23.54 ± 1.11	0.013^a^
Posterior	3	0	0	0.015^a^
staphyloma	4	0	4	
ERM	3	0	4	0.088
LHEP				0.414
ILM peeling area (PD)				
(mean ± SD)	3.92 ± 0.49	3.67 ± 0.76	3.68 ± 0.71	0.744
BCVA improvement (mean ± SE)	0.45 ± 0.13	0.37 ± 0.15	0.17 ± 0.13	0.630

*AL* Axial length, *ERM* Epiretinal membrane, *LHEP* Lamellar hole associated epiretinal proliferation, *ILM* Inner limiting membrane, *PD* Papillary diameter, *BCVA* Best corrected visual acuity, *SE* Standard error

^a^Indicates a statistical significant difference between (*P* ≤ 0.05)

## Discussion

In this study, we used multiple imaging modes of spectral domain OCT to observe the inner retinal layer changes after ILM removal in patients with MHs. Inner retinal defects are frequently [1–6] seen after ILM peeling (43–100%), which was confirmed by our study (68%). On en face OCT ILM layer images, inner retinal defects can be seen as multiple dots distributed on the inner surface of the RNFLs, appearing darker than the surrounding area. On reviewing the en face OCT images, we were able to identify 3 distribution patterns of the dots: papillomacular bundle-dominated, scattered, and temporal raphe-spared. The dark dots on the ILM layer images corresponded to the dimples on the inner retinal surface noted on the cross-sectional OCT images. This is similar to the reports by Mitamura [2] and Ito [3], who found dimples in the RNFL using B-scans of OCT corresponding to each stria of the DONFL. They found that the depths of these dimples were limited to the RNFL thickness. However, we found ganglion cell layer thinning was concomitant with the dimples in some eyes, which was also found by Spaide [6]. Ganglion cell layers play a significant role in visual function. In order to determine whether retinal function was adversely influenced, we compared the BCVA and retinal average sensitivity thresholds detected by MAIA, and found no difference between the 2 groups. The outcome confirmed the findings of previous studies [1, 3, 6, 8]. However, some studies did find abnormal scotomata and reduced retinal sensitivity after surgery for MH [15, 16]. As many as 35–50% of retinal ganglion cells can be lost before a visual function deficiency is detected [17–19]. Based upon our results, we speculate that retinal ganglion cell losses occurring with inner retinal dimples may not reach the number needed to compromise visual function or our follow-up durations were not long enough to detect these changes. Another explanation was that these findings were not due to ganglion cell loss because Müller cell bodies are also located in the ganglion cell layer. Histopathologic studies [20–22] have shown that the peeled ILM contains Müller cell footplates, which would lead to Müller cell degeneration. Therefore, this may result in the thinning of the ganglion cell layer.

Müller cell footplates are reported to play a significant part in maintaining the homeostasis of the retinal milieu [23, 24]. The damaged footplate function after ILM peeling may be followed by secondary physiologic derangements of the inner retina [6]. Thus, the effects of trauma to the Müller cells, along with their regenerative growth after surgical removal of the ILM, could explain the formation of inner retinal dimples [6]. The relationships of ERM and LHEP, which are involved in the proliferation of glial cells, with inner retinal defects formation were evaluated. However, in this study, eyes with LHEP or conventional ERM in the inner retinal dimples group were not significantly different from eyes in the control group. The results suggest that the formation of inner retinal dimples may be influenced by several factors apart from Müller cells, that are yet to be explored. In addition, LHEP was found adjacent to the edges of macular holes, far away from the ILM peeling boundaries which were close to the vascular arcade in most eyes in this study. Therefore, a large portion of ILM peeling area was not covered by LHEP. Due to the small sample size of our study, the results need to be verified by further studies.

In this study, we failed to identify a relationship between inner retinal dimples formation and different vitreomacular interfaces due to high myopia in the current study. Sakimoto et al. [25] described inner retinal defects after ILM peeling in high myopic eyes and found that different ILM-retinal adhesions in different fundus areas influenced the development of inner retinal defects. However, only one eye with ILM detachment was included in our study, which had no evident inner retinal dimples observed on both OCT scans (en face and B-scan) after surgery. However, we did found that a large portion of the eyes with longer axial length and posterior staphyloma formed “scattered” inner retinal dimples. These inner retinal defects were related to significant thinning of inner retinal layers. We speculated the globe deformation of posterior staphylomas in highly myopic eyes led to different ILM retina adhesion [26, 27], thus the different type of inner retinal defects. In addition, the choroid plays an important role in offering nutrient and oxygen to the retina in the macular area [28]. Therefore, we speculate that the nerve fiber layer was susceptible to surgical manipulation in the high myopic eyes due to myopic chorioretinal atrophy [26, 29]. Further, the decreased level of neutrophic factor in high myopic eyes [30] may compromise healing after ILM peeling in these eyes.

In 5 cases, we observed an apparent progression of inner retinal dimples, which appeared on en face ILM layer images as increasing numbers and sizes of dark dots. The inner retinal defects began in the central macular area and spread to the surrounding area. We speculated that these dynamic changes of inner retinal defects are because the retinal nerve fiber bundles surrounding the arcade which is located near the ILM peeling boundary are the thickest, and it takes some time before any changes can occur. Despite the apparent progression, the defects remained in the ILM peeling area. We believe after ILM peeling, the inner retinal may experience a continuous degeneration. However, this differs from the findings of Alkabes et al. [5] They also used en face OCT to observe these postoperative changes and believed that they were stable over time. The cause of these dynamic changes needs to be explored further.

The longest follow-up was 3 years, with no spontaneous resolution of the defects. Although we obtained B-scan OCT images through the radial and horizontal line imaging modes together, it was not feasible to observe the distribution form, and it was also difficult to analyze the dynamic changes in the inner retinal dimples on these cross-sectional OCT images. Therefore, monitoring inner retinal dimples after ILM peeling using en face and B-scan OCT imaging is far more advantageous than performing the same evaluation using B-scan OCT imaging alone. On some of the reference images, we could even distinguish the ILM peeling area readily, which is difficult to find on funduscopic examination. This information could be useful in helping surgeons make individualized surgical plans in cases of failed MH closure after previous ILM peeling: enlarged peeling and insertion of ILM [31] and transplantation of lens capsular flap [32] in patients with and without enough remnant ILM, respectively. We therefore drew the conclusion that the different images obtained by various OCT imaging modes can provide detailed information about the inner retinal defects.

The primary limitations of our study include the retrospective design, the small sample size, and the short observation time. Moreover, there was not enough high myopic eyes included in this study because the poor segmentation performance in these eyes. In addition, microperimetry was only performed in a limited number of eyes, so the comparison of the average sensitivity threshold between eyes with different patterns of inner retinal defects were not conducted. Studies with prospective designs and larger sample sizes are needed to analyze the formation of inner retinal defects, and determine if they affect retinal function.

## Conclusions

In conclusion, inner retinal dimples were frequently found after ILM peeling. En face OCT imaging combined with B-scan OCT imaging is highly recommended to evaluate inner retinal dimples after ILM peeling, due to the comprehensive information that these scans can provide to clinicians. High myopic eyes might develop more severe, “scattered” inner retinal defects after ILM peeling. Inner retinal dimples after surgery do not seem to compromise visual function in the short-term.

## Abbreviations

BCVA: Best corrected visual acuity
DONFL: Dissociated optic nerve fiber layer
ERM: Epiretinal membrane
FTMH: Full-thickness macular hole
ILM: Inner limiting membrane
LHEP: Lamellar hole-associated epiretinal membrane
LMH: Lamellar macular hole
logMAR: Logarithm of the minimum angle of resolution
MH: Macular hole
OCT: Optical coherence tomography
PPV: Pars plana vitrectomy
